# Spanish adaptation of the quality in psychiatric care-outpatient (QPC-OP) instrument community mental health patients’ version: psychometric properties and factor structure

**DOI:** 10.1186/s12912-022-01094-8

**Published:** 2022-11-08

**Authors:** Manuel Tomás-Jiménez, Juan Francisco Roldán-Merino, Sara Sanchez-Balcells, Agneta Schröder, Lars-Olov Lundqvist, Montserrat Puig-Llobet, Antonio R. Moreno-Poyato, Marta Domínguez del Campo, Maria Teresa Lluch-Canut

**Affiliations:** 1 Parc Sanitari Sant Joan de Déu. Sant Boi de Llobregat, Barcelona, Spain; 2 Mental Health Department, Campus Docent Sant Joan de Déu-Private Foundation, University of Barcelona, C/ Sant Benito Menni, 18-20, 08830 Sant Boi de Llobregat, Spain; 3 University Health Care Research Center, Faculty of Medicine and Health, Örebro University, Örebro, Sweden; 4 Department of Nursing, Faculty of Health Care and Nursing, Norwegian University of Science and Technology (NTNU), Trondheim, Norway; 5 Public Health, Mental Health and Maternal-Infant Nursing Department, Nursing College, University of Barcelona, Health Sciences Campus Bellvitge, Hospitalet de Llobregat, Barcelona, Spain

**Keywords:** Community care, Psychometric properties, Patients’ perspective, Quality of care, Psychiatric care

## Abstract

**Background:**

Health systems in the field of mental health are strongly committed to community models that allow patients to be attended in their own environment. This helps them to maintain their family and social ties while trying to avoid costly hospital admissions. The patients’ perspective is a key component in the assessment of the quality of psychiatric care and can even determine their adherence to the devices where they are treated. However, there are few instruments with adequate psychometric properties for the evaluation of the quality of psychiatric care in community mental health. The *Quality in Psychiatric Care – Outpatient (QPC-OP)* instrument has adequate psychometric properties to assess the quality of psychiatric care from the patients’ perspective. The aim of this study was to adapt and validate the Spanish version of the *QPC-OP* instrument.

**Methods:**

A translation and back-translation of the instrument was carried out. To examine its psychometric properties, the instrument was administered to 200 patients attending various community mental health services. To assess test-retest reliability, the instrument was readministered after 7-14 days (*n* = 98).

**Results:**

The Confirmatory Factor Analysis revealed a structure of 8 factors identical to the original version, with an adequate model fit. The internal consistency coefficient (Cronbach’s alpha) was 0.951. The intraclass correlation coefficient was 0.764 (95% IC: 0.649 – 0.842), and higher than 0.70 in 5 of the 8 factors. Additionally, an EFA was performed and revealed that the instrument could behave in a unifactorial or four factor manner in the sample analyzed.

**Conclusions:**

Results show that the Spanish version of the *QPC-OP* instrument is valid and reliable for the assessment of quality of psychiatric care in the community setting.

## Background

At the international level, it has been observed that investing in the provision of services related to mental health (prevention, intervention and health promotion) produces benefits in terms of health and social factors. In addition, economic costs associated with mental health problems are reduced [1]. Current trends support a community model of mental health care which replaces long, expensive hospital stays with treatment of patients in their community so that they do not lose their family and social connections [2–4]. These services should be developed following a recovery ethic and should focus on the person and their autonomy, along with the therapeutic relationship [2, 5]. Coordinated, comprehensive care of people with mental health problems in the community where they live is associated with a perception of greater satisfaction in this population [6].

Wilson and Daly, promoted the move away from the medical model of care to encourage a spirit of recovery in which patients and caregivers are recognized as equal partners in decision-making and certain aspects of planning, such as the provision of mental health services [7]. A focus on individual preferences and patient participation in decisions is vital to the development of a recovery-oriented system [8, 9].

Patients’ perceptions are relevant indicators when assessing interventions and the quality of the health service in question [10, 11] and, in some cases, the capacity to respond to their expectations is considered a performance parameter of the health system itself [12]. The area of community mental health is no exception, given that patients’ perceptions of the quality of psychiatric care received have become an important component in assessments [13] and have even been used as an indicator of quality [14].

Quality of care is a fundamental component of the right to health and essential in ensuring equity and dignity among patients of health services [15]. Services that meet quality of care criteria can lead to expected health outcomes [16] and better quality of life [17]. However, deficits in quality of care can contribute to dissatisfaction, lack of adherence and even an increase in patient mortality [16].

There is no widely accepted definition of quality of care. This term refers to a multidimensional concept [18] that is perceived by the mental health patient as positive [19]. As distinct from the term satisfaction, quality of care includes the perspectives of all parties involved [20]. It is important to conduct research into those factors that may affect the rating of psychiatric care received by patients, given that a better understanding of the factors that have a negative impact on such ratings will allow both professionals and the administration to improve the patients’ experience [21].

There is a lack of intercultural studies that compare the perceptions of professionals and patients on the quality of care [22] and this is mainly due to the very few standardized instruments with adequate psychometric properties available for the assessment of this care [23].

A recent systematic review reported that there is a scarcity of instruments for the evaluation of satisfaction and quality of psychiatric care that present an acceptable validation process and adequate psychometric properties [24]. Some instruments are based on the professionals’ point of view on care quality [25, 26], despite the fact that the perceptions of professionals and patients frequently differ on what constitutes good quality of care [27]. In fact, there are authors who question the validity of assessing the quality of psychiatric care without taking the patients’ perspective into account [28] as these patients are an essential component in the development of measurement instruments [29, 30].

The systematic review mentioned above highlights two instruments within the field of community mental health [24]. One is the Psychiatric Out-Patient Experiences Questionnaire (POPEQ) which assesses experiences solely from the patients’ point of view [29]. The other instrument is Quality in Psychiatric Care – OutPatient (QPC-OP) [31], an instrument of Swedish origin that evaluates the quality of psychiatric care in the community environment from the perspectives of both professionals (QPC-OP Staff) and patients (QPC-OP). This instrument forms part of a battery of Quality in Psychiatric Care tools which assess the quality of psychiatric care in community [31], hospital [32] and forensic settings [33]. The definition of quality used to create the instrument was developed from a phenomenographic study [19], assessed for apparent validity in a pilot study and tested in a sample of patients admitted to psychiatric hospitalization units in Sweden [30].

This study is part of a wider international project to adapt the QPC-OP patient version of the instrument in different countries, test its psychometric properties and the equivalence in dimensionality of the different versions by language, and describe and compare the quality of psychiatric care in the community setting in various countries.

## Methods

### Aim

The aim of the present study was to adapt the patient version of the QPC-OP instrument into Spanish and analyze its reliability and validity.

### Design

The study has a descriptive cross-sectional psychometric research design and was carried out in two phases. The first phase involved the translation and adaptation of the QPC-OP instrument into Spanish and, in the second phase, the metric properties of the Spanish version of the QPC-OP instrument were analyzed.

### Participants and study setting (sample size)

The sample consisted of 200 patients from community health facilities who met the following inclusion criteria: older than 18 years, having a diagnosis of mental disorder, being followed up in community mental health facilities at the time of the study, and agreeing to participate voluntarily in the study. Exclusion criteria were: inability to understand or speak Spanish, significant cognitive impairment, organic disorder and/or intoxication due to drug use at the time of assessment. Consecutive, non-probabilistic sampling was used.

Data collection was carried out between February, 2020 and March, 2022. The long period of data collection was due to the difficulty in accessing patients in the context of the COVID situation, which affected community mental health centers at the organizational level and required health priorities to be modified.

Calculation of the sample size was carried out based on internal consistency, temporal stability and construct validity. To estimate internal consistency, the recommendations of Streiner, Norman & Cairney (2015) were followed, which consider that between 5 and 20 individuals should be included for each item that makes up the scale [34]. In this study, it was agreed to include a minimum of 5 individuals. On the other hand, the authors considered that the minimum number required to conduct the confirmatory factor analysis was 200 participants [35, 36].

To analyze temporal stability, it was estimated that a minimum of 61 participants would be needed to detect an intraclass correlation coefficient (ICC) of around 0.70 between two administrations, assuming a confidence level of 95% and a power of 80% in a bilateral comparison [37].

### Variables and sources of information

As indicated, the QPC-OP instrument assesses the quality of psychiatric care in the community setting from the patients’ perspective and the internal consistency of the whole instrument in the original version shows adequate results (α = 0.95) [31].

This instrument is made up of 8 factors that represent the quality of psychiatric care in the community setting and the internal consistency of each factor in the original version is shown below. The encounter factor (α = 0.94) represents the interpersonal relationship between the patient and the professional, in which the patient evaluates the degree of empathy, respect, concern and listening that the professional demonstrates. The participation-empowerment (α = 0.92) and participation-information (α = 0.90) factors show the degree of participation in care from the patient’s perspective, as well as whether they consider that they have the essential information to be able to make decisions about their care. The discharge factor (α = 0.66) shows the continuity of care provided by the community mental health center, while the support factor (α = 0.85) shows the support that patients receive from professionals in relation to the stigma associated with mental illness. The environment factor (α = 0.76) represents the degree of safety that patients feel within the center. On the other hand, the next of kin factor (α = 0.65) shows the degree of respect and participation in the care that the close relatives of the patients have. Finally, the accessibility factor (α = 0.79) evaluates the difficulties in contacting the center and the patient’s assigned professionals.

It consists of a total of 30 items distributed across 8 factors as follows: Encounter (6 items), participation-empowerment (3 items), participation-information (5 items), discharge (3 items), support (4 items), environment (3 items), next of kin (2 items) and accessibility (4 items). Each item begins with the words “I feel that…” and is scored on a Likert type scale with 4 response options ranging from 1 (totally disagree) to 4 (totally agree), with a further “not applicable” option for each one if necessary. Scores can be obtained globally or by factors; the maximum global score is 120 points and the minimum 30 points, such that a high score on each factor or on the instrument overall indicates a good perception of quality of psychiatric care on the part of patients. On the other hand, a low score justifies the need for an intervention to make improvements in identified areas.

In addition, other variables related to the sample’s sociodemographic characteristics were collected: age, sex, nationality, educational level, and the community facility that the patient attended.

### Procedure

The translation and back-translation were carried out in accordance with the Standards for Educational and Psychological Testing [38].

The original version of the instrument was translated into Spanish by two native-speaker professional translators who were unaware of the instrument or the aims of the study. A group of experts including nurses, psychologists and psychiatrists reviewed the translation and agreed on the first version of the instrument in Spanish language. Consecutively, the Spanish version was back-translated into the original language to confirm its concordance with the original Swedish version. The original authors of the QPC-OP then examined the back-translation and compared it with the original, finding no discrepancies requiring modifications. A pilot test was carried out in 30 patients with the aim of assessing clarity and comprehension of the items, along with the time required to complete the instrument. Following debriefing, it was not necessary to make any changes to either content or format.

After obtaining the final version of the instrument, the community mental health services nurses recruited the participants after their appointment at the center that day using consecutive non-probabilistic sampling. The patients independently filled out the instruments, consulting the nurses if queries arose. The patients were scheduled within the following 7-14 days to complete the instrument again in order to determine the temporal stability of the instrument.

### Statistical analysis

#### Construct validity

Construct validity was analyzed through confirmatory factor analysis (CFA) with parameters estimated using the least squares method with a polychoric correlation matrix. This method has the same properties as the maximum likelihood method, despite the fact that the criteria were less strict than the normal ones. It is mainly used to measure ordinal items [39].

The following fit indices were calculated to determine the overall fit of the model:

Bentler Bonnet Normed Fit Index (BBNFI), Bentler Bonnet Non-Normed Fit Index (BBNNFI), the Goodness-of-Fit Index (GFI), the Adjusted Goodness-of-Fit Index (AGFI), the Comparative Fit Index (CFI), the Root Mean-Square Residual (RMR), the Standardized Root Mean-Square Residual (SRMR), the Root Mean Square Error of Approximation (RMSEA), the chi-squared goodness-of-fit test and the ratio between chi-squared and the degrees of freedom (χ2/df). The criteria for a good fit were an X2/df ratio < 3 and BBNFI, BBNNFI, GFI, AGFI and CFI values close to 0.90 [40–42], and RMR, SRMR and RMSEA values lower than 0.08 [43, 44].

#### Reliability

Cronbach’s Alpha coefficient was used to assess the internal consistency of the instrument globally and for each of its dimensions. Reliability values higher than 0.70 [34] were considered adequate.

Temporal stability or test-retest was assessed after 7-14 days through the intraclass correlation coefficient (ICC) in a sample of 98 patients. Coefficient values ranged between 0 and 1. A value equal to or greater than 0.70 was considered an indicator of good agreement [34].

The statistical program SPSS Statistics version 28 was used for data analysis, EQS version 6.3 was used for confirmatory factor analysis (CFA) [45] and Factor software for parallel analysis [46].

## Results

### Participant characteristics

A total of 200 patients with a mean age of 46.15 ± 13.74 years took part in the study; 49.7% male and 50.3% females. Some 92% of the participants were of Spanish nationality and the rest were of various ethnic origin. Regarding educational level, 11.8% had not completed primary education while 21% had completed primary and 17.9% had completed secondary education. A total of 32.8% had completed professional/baccalaureate/equivalent and the remaining 16.4% had completed higher education or a university course.

### Construct validity

#### Confirmatory factor analysis (CFA)

Confirmatory factor analysis was used to verify the internal structure of the instrument, in which an 8-dimension model identical to the structure of the original version was proposed. Table 1 shows the fit of the model. All indices showed a reasonable fit.

**Table 1:** Goodness-of-fit indices for the confirmatory model Spanish QPC-OP

Index	Value
BBNFI	0.775
BBNNFI	0.831
GFI	0.982
AGFI	0.977
CFI	0.853
RMR	0.036
SRMR	0.057
RMSEA	0.084
Cronbach’s alpha	0.951
Goodness of fit test	χ2 = 900.444; df = 377; *p* < .0001
Adjustment reason	χ2/df = 2.38

*BBNFI* Bentler Bonnet Normed Fit Index, *BBNNFI* Bentler Bonnet Non-Normed Fit Index, *GFI* Goodness-of-Fit Index, *AGFI* Adjusted Goodness-of-Fit Index, *CFI* Comparative Fit Index, *RMR* Root Mean-Square Residual, *SRMR* Standardized Root Mean-Square Residual, *RMSEA* Root Mean Square Error of Approximation, *df* Degrees of freedom

**Fig. 1:**
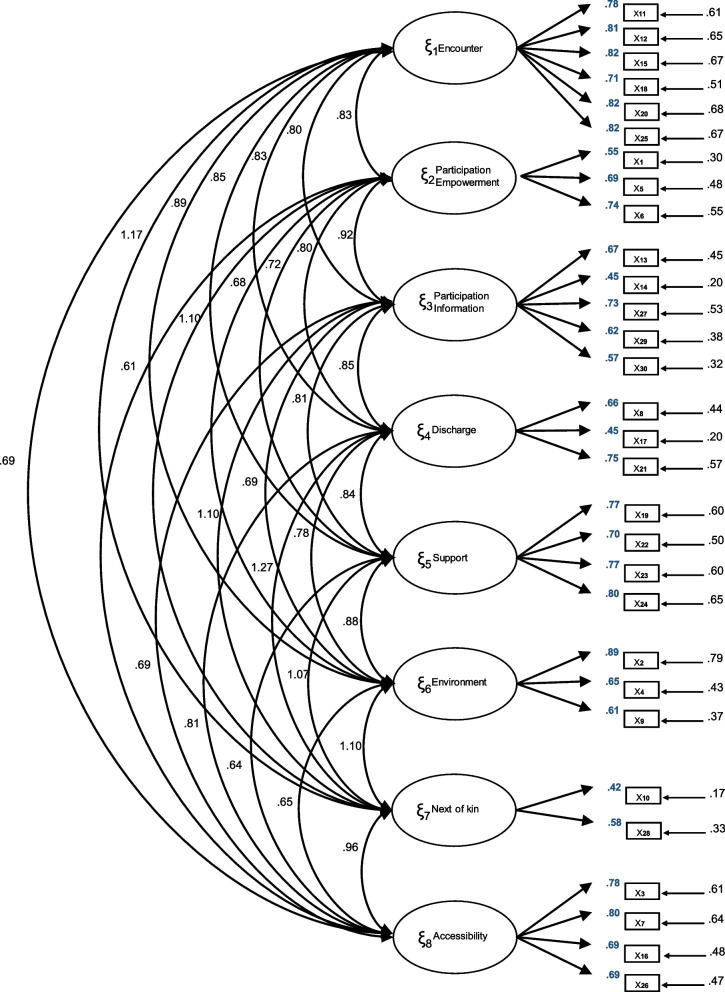
Factor loadings derived from the least squares estimation (least squares). Confirmatory factor analysis (λij)

All item loadings were equal to or greater than 0.50 with the exception of items 14 (0.45), 17 (0.45) and 10 (0.42). The correlations between the factors of the Spanish QPC-OP instrument are shown in Fig. 1.

It was also decided to carry out a parallel analysis with an Exploratory Factor Analysis (EFA) in this study to determine the factorial model with the best fit to the Spanish sample analyzed. To conduct the EFA, its suitability was previously analyzed using the Bartlett sphericity test and the Kaiser Mayer test (KMO). The value of the KMO was 0.955, which was deemed acceptable to proceed with the EFA, and the significance level of the Bartlett sphericity test was χ2 = 2166.3; df = 435; *p* < .0001. For the extraction of the factors, three basic premises were taken into account: (1) the graphical inspection of the Scree plot [47]; (2) Kaiser’s rule, which recommends retaining components with values equal to or greater than 1 [48], and (3) the optimal implementation of Horn’s parallel analysis. This is the most suitable method to identify the number of factors [49].

The adjustment method selected for factor extraction was Weighted Least Squares with corrective adjustment statistics for the mean and variance. For the rotation of factors, the robust promin rotation was used [50].

Four factors had autovalues greater than 1, which explains 70.2% of the variance. However, the Scree plot and the results of the parallel analysis indicated 1 value for which the real data autovalue exceeded the random data autovalues. Some 56.7% of the variance is explained by a single factor.

To assess whether the instrument could be considered essentially one-dimensional, Explained Common Variance (ECV) and Unidimensional Congruence (UniCo) indices were also calculated to assess the degree of dominance of the general factor or the closeness to unidimensionality.

Table 2 shows the fit indices for the unifactorial and the four-factor model respectively.

**Table 2:** Goodness-of-fit indices of the exploratory unifactorial and the four-factor to the model

	Unifactorial		Four factor	
Index	Value	95% confidence interval	Value	95% confidence interval
GFI	0.985	0.980 - 0.991	0.995	0.989 - 0.996
AGFI	0.984	0.979 - 0.990	0.993	0.985 - 0.995
CFI	0.996	0.988 - 1.011	0.995	0.989 - 0.995
RMSEA	0.034	0.007- 0.058	0.044	0.010- 0.050
Goodness of fit test	χ2 = 497.883; df = 405; P < 0.001		χ2 = 446.587; df = 321; *P* < 0.001	
Reason for fit	χ2 / df = 1.22		χ2 / df = 1.39	

*GFI* Goodness-of-Fit Index, *AGFI* Adjusted Goodness-of-Fit Index, *CFI* Comparative Fit Index, *RMSEA* Root Mean Square Error of Approximation, *df* Degrees of freedom

The ECV index essentially measures the proportion of common variance of the item scores that can be explained by the first canonical factor (i.e., the factor that explains most of the common variance).

The value of the UniCo index was 0.988 (IC 95%: 0.979 – 0.995) and an ECV value of 0.937 (IC 95%: 0.932 – 0.953) was obtained. For the instrument to be considered as essentially one-dimensional, the ECV values should be greater than 0.85 [51] and those of the UniCo index should be greater than 0.95 [52].

Table 3 shows the factor loads of each item following the four-factor model.

**Table 3:** Loading matrix for the exploratory four-factor analysis solution

Item	Factor 1	Factor 2	Factor 3	Factor 4
**1**				0.866
**2**			0.614	
**3**		0.825		
**4**			0.892	
**5**				0.913
**6**	0.627			
**7**		0.404		
**8**				0.697
**9**			0.927	
**10**				0.381
**11**				0.728
**12**				0.694
**13**				0.789
**14**				0.306
**15**			0.524	
**16**		1.055		
**17**		0.316		
**18**				0.798
**19**			0.527	
**20**			0.475	
**21**			0.448	
**22**			0.704	
**23**			0.713	
**24**			0.695	
**25**				0.471
**26**		0.469		
**27**	0.748			
**28**			0.519	
**29**	0.680			
**30**	0.908			

#### Reliability

Following the original factor structure, Cronbach’s alpha internal consistency coefficient for the whole instrument was 0.951, reaching values higher than 0.70 in five of the eight factors (Table 4). In the factors *F2. Participation–Empowerment, F4. Discharge and F7. Next of kin*, Cronbach’s alpha values of 0.697, 0.650 and 0.373 were obtained, respectively.

**Table 4:** Spanish QPC-OP. Cronbach’s alpha coefficient, test-retest ICC (*n* = 98)

Instrument factors	Cronbach’s alpha	ICC (CI 95%)	SME
F1. Encounter	0.913	0.755 (0.635 - 0.836)	1.017
F2. Participation - Empowerment	0.697	0.589 (0.387 - 0.724)	1.065
F3. Participation – Information	0.738	0.689 (0.535 - 0.791)	1.562
F4. Discharge	0.650	0.710 (0.567 - 0.806)	1.106
F5. Support	0.850	0.780 (0.669 - 0.853)	0.895
F6. Environment	0.761	0.727 (0.591 - 0.817)	0.920
F7. Next of kin	0.373	0.713 (0.569 - 0.808)	1.008
F8. Accessibility	0.827	0.684 (0.529 - 0.788)	1.116
**Total**	**0.951**	**0.764 (0.649 - 0.842)**	**3.334**

*ICC* Intraclass Correlation Coefficient, *CI* Confidence Interval, *SME* Standard Measurement Error

The ICC analysis demonstrated that test-retest reliability was 0.764 (95% IC: 0.649 - 0.842; *n* = 98), and this value was higher than 0.70 in all instrument factors except F2, F3 and F8, whose values were 0.589, 0.689 and 0.684.

In the parallel analysis, the reliability of the factors following the 4-factor model shows Cronbach’s Alpha values greater than 0.70 in all factors, as can be seen in Table 5. The ICC shows values higher than 0.70 in all factors except Factor 2, with a value of 0.669 that could be considered adequate.

**Table 5:** Spanish QPC-OP four-factor model. Cronbach’s alpha coefficient, test-retest ICC (*n* = 98)

Instrument factors	Cronbach’s alpha	ICC (CI 95%)	SME
F1	0.822	0.709 (0.566 - 0.805)	1.197
F2	0.803	0.669 (0.505 - 0.778)	1.384
F3	0.924	0.819 (0.729 - 0.878)	1.644
F4	0.875	0.736 (0.607 - 0.823)	1.903
**Total**	**0.951**	**0.764 (0.649 - 0.842)**	**3.334**

*ICC* Intraclass Correlation Coefficient, *CI* Confidence Interval, *SME* Standard Measurement Error

## Discussion

The aim of this study was to adapt the Quality in Psychiatric Care Outpatient (QPC-OP) instrument into Spanish and analyze its reliability and validity.

To generate the adapted Spanish version of the QPC-OP instrument, a translation/back-translation process was carried out. Other studies of QPC instruments [53–62] have obtained versions in other languages through a similar process. The results in this phase were satisfactory and no difficulties were observed with respect to degree of comprehension or administration of the instrument.

Regarding psychometrics, construct validity (CFA), internal consistency and temporal stability (test-retest) values were adequate.

The confirmatory factor analysis (CFA) carried out indicated that the Spanish version has the same 8 quality of psychiatric care factors as the original Swedish QPC-OP version [31] and no items required modification. In addition, an EFA was performed to determine the factorial model with the best fit to the Spanish sample analyzed, revealing that it could behave in a one-dimensional manner. The EFA also demonstrated an adequate fit for the model with the 4-factor structure in the sample analyzed.

Factor 1 in this new model groups items that make up dimensions F2.Participation-empowerment and F3.Participation-information of the original structure. This is reasonable as it brackets items related to users’ participation in their care.

Furthermore, factor 2 of the four-factor structure fully groups the items that make up F8.Accessibility of the original structure and an item from F4.Discharge. This makes sense as the item belonging to F4.Discharge measures the perception of the user regarding help received from professionals to look for work or other occupations and the items that form F8.Accessibility represent the potential difficulties that users perceive in contacting either the professionals or the center.

Separately, factor 3 entirely consists of items from F5.Support and F6.Environment, along with some items from F1.Encounter, F4.Discharge and F7.Next of Kin. This is logical as these items assess the user’s perception of safety and respect (not doing harm to oneself or others, feeling safe with the other users and with the center itself, professionals’ respect for users and their relatives, etc.).

Finally, factor 4 seems to include some items from F1.Encounter, F2.Participation-empowerment, F3.Participation-information, F4.Discharge and F7.Next of kin of the original structure. These items assess the user’s perception of treatment received (interest shown, understanding and concern for users’ feelings and care received) as well as respect in decision-making.

Analysis of the reliability of the instrument with the 8 original factors was performed using Cronbach’s alpha coefficient. Globally, the instrument showed a Cronbach’s alpha of 0.951, and in 5 of 8 factors values greater than 0.70 were obtained; an adequate value according to Nunnally and Berstein [63]. The global Cronbach’s alpha is identical to that of the original instrument [31], with a higher value than the Spanish professional versions in both the hospital and community settings [53, 59]. This value is also higher than the Spanish version for patients in the hospital setting [58], Indonesian hospital versions [56, 57], the original hospital version for professionals [64], and the community version for Norwegian professionals [65]. However, it showed a somewhat lower value than other versions of the QPC instrument [32, 33, 54, 66, 67]. Of all the factors, F7 (Next of Kin) showed the lowest reliability, as was the case in the original Swedish version [31]. This may be due to the fact that this factor consisted of only two items and despite the fact that these results were not optimal, it was considered a priority to preserve the original structure of the instrument in the Spanish version.

Temporal stability or test-retest was calculated within the original 8-factor structure. This value was not determined in either the original Swedish QPC-OP version [31] or in other original community, patient’s hospital or either forensic QPC versions [32, 33, 66–68] or their translated versions [54–57, 65], with the exception of the original hospital version for professionals [64], Spanish hospital versions [53, 58] and the Spanish community version for professionals [59]. The ICC for the total instrument and for each of the factors demonstrated good agreement [34, 37], with values higher than 0.70 except in F2 (0.589), F3 (0.689) and F8 (0.684). This global value is lower than that of the original hospital version for professionals [64] and that of the rest of the Spanish versions although it should be pointed out that these also showed values lower than 0.70 in some factors. Specifically, the Spanish hospital versions showed values lower than 0.70 in F6. Discharge [53, 58], while in the Spanish community version for professionals [59], it was F5. Support that showed a lower value than we would have preferred. This was not the case in the original hospital version for professionals, where all the factors showed values over 0.70 [64]. However, the four factor structure presented based on the EFA showed greater reliability in each of the dimensions analyzed.

As a limitation we should stress that it was not possible to assess the sensitivity to change or predictive validity due to the cross-sectional design of the study design. A potential limitation could result from the use of a traditional method to calculate the sample size although, given the complexity of the sample, a sample size of 200 participants was established to carry out the confirmatory factor analyses. It may be the case that the sample is not entirely representative of the study population although the participants were selected from a wide geographical area so that the results can be extrapolated. For the Spanish sample, the unidimensionality or the four dimensions model of the instrument can be considered, although studies with a larger sample size are required to determine whether the one or four-dimensional behavior is maintained. In fact, a larger sample size is highly recommended for further research in general. Another limitation, regarding recruitment, was due to the difficulties arising from the COVID-19 pandemic at health facilities. Future research should consider the collection of other variables that could influence the patient’s perception of quality in psychiatric care to ensure that all such factors are taken into account, as well as the type I intensity of the mental health disorder. Longitudinal designs are recommended for future research to assess sensitivity to change. The data necessary to allow calculation of convergent validity were not collected and this determination is recommended in future research. These limitations should be taken into account in the design of future studies.

## Conclusions

The Spanish version of the QPC-OP instrument is a simple, useful tool for the measurement of various aspects of quality of psychiatric care from the perspective of patients in the community mental health setting. Its 8-factor structure and its psychometric properties are consistent and in accordance with the original version, thus allowing this instrument to be used to assess the quality of services provided from the perspective of Spanish-speaking patients. However, the model that best fit the Spanish sample analyzed revealed a unifactorial or four-factor structure that should be considered in future studies. We would like to emphasize the importance of understanding the perception of psychiatric care received in the community setting given the mainly voluntary connection that patients have with these services.

### Implications for practice

Having an instrument that assesses the quality of psychiatric care created from the perspective of patients is of great value in improving the quality of care in outpatient mental health services. In addition, this instrument can be useful in assessing the impact of new interventions or organizational changes carried out in these services from the perspective of mental health care recipients. Detecting strong points and areas for improvement in services can guide professionals in new strategies to improve the quality of the psychiatric care they provide, thus reinforcing the links between patients and both professionals and health devices while ensuring quality health care. However, the joint use of the Spanish QPC-OP with its complementary instrument in professionals (Spanish QPC-OPS) [59] should be considered to obtain both perspectives simultaneously when evaluating the quality of psychiatric care.

## Data Availability

The datasets used and/or analysed during the current study are available from the corresponding author on reasonable request.
